# Potential of *Aframomum melegueta* and *Xylopia aethiopica* Against *Taenia* spp.: Plant-Based Remedies as Novel Anthelmintics

**DOI:** 10.3390/ph18050749

**Published:** 2025-05-19

**Authors:** Fekandine V. Douti, Gnatoulma Katawa, Kathrin Arndts, Fagdéba D. Bara, Essimanam R. Awesso, Simplice D. Karou, Achim Hoerauf, Manuel Ritter

**Affiliations:** 1 Unité de Recherche en Immunologie et Immuno-Modulation (UR2IM), Laboratoire de Microbiologie et de Contrôle de Qualité des Denrées Alimentaires, École Supérieure des Techniques Biologiques et Alimentaires (ESTBA), Université de Lomé, Lomé 01 BP 1515, Togo; doufekandine@gmail.com (F.V.D.); fagdeba.bara@gmail.com (F.D.B.); roseawesso@gmail.com (E.R.A.); simplicekarou@hotmail.com (S.D.K.); 2 Institute for Medical Microbiology, Immunology and Parasitology (IMMIP), University Hospital Bonn (UKB), 53127 Bonn, Germany; kathrin.arndts@ukbonn.de (K.A.); hoerauf@uni-bonn.de (A.H.); 3 German-West African Centre for Global Health and Pandemic Prevention (G-WAC), Partner Site Bonn, 53127 Bonn, Germany; 4 German Centre for Infection Research (DZIF), Partner Site Bonn-Cologne, 53127 Bonn, Germany

**Keywords:** *Aframomum melegueta*, *Xylopia aethiopica*, fractions, anthelminthic, *Taenia* spp.

## Abstract

**Background/Objectives:** Taeniasis, a zoonotic infection, is a common foodborne disease. Niclosamide and praziquantel have proven to be effective in treating it, but the use of the same drugs can lead to resistance, so alternative drugs need to be explored. This study investigated the anthelmintic potential of derived fractions from hydroethanolic extracts (HEs) of *Aframomum melegueta* (AM) and *Xylopia aethiopica* (XA), two medicinal plants known for their diverse bioactive properties. **Methods:** AM-HE fractions (dichloromethane fraction (DCMF), ether fraction (EF), aqueous fraction (AF)) and XA-HE fractions (chloroform fraction (CF), ether fraction (EF), and aqueous fraction (AF)) were used, and in vitro anthelmintic activity was assessed against *Taenia* spp. by using an adult motility assay for the worm’s paralysis time determination. The parasiticidal and parasitostatic activity was also tested on *Taenia* spp. adult worms. Cell viability was further evaluated using propidium iodide (PI) staining, with albendazole (20 mg/mL) as the reference drug. **Results:** The three fractions of each plant exhibited significant, dose-dependent anthelmintic activity, with AM-HE and XA-CF showing the greatest effects at 20 mg/mL. AM-EF demonstrated significant activity at 0.4% and 0.8%. Irreversibility tests revealed that most of the treated worms remained paralysis, except those exposed to the AF of both plants. PI staining confirmed the dose-dependent mortality of *Taenia* cells treated with HE, DCMF, and AF of AM. **Conclusions:** These results underscore the potential of AM and XA extracts and fractions as alternative treatments for helminth infections. Further, in vivo studies are warranted to confirm their safety and therapeutic efficacy.

## 1. Introduction

Taeniasis is an intestinal infection that affects both humans and animals, caused by the adult stages of tapeworms. These parasites belong to the class of *Cestoda*, the family of *Taeniidae*, and the genus *Taenia* [1]. Three species are known to infect humans: *Taenia asiatica* (Asian tapeworm) [2], *Taenia saginata* (beef tapeworm), and *Taenia solium* (pork tapeworm). While *T. asiatica* is restricted to Asia, *T. saginata* and *T. solium* have a global distribution.

The transmission of taeniasis is influenced by several environmental factors, including the dispersal of parasite eggs in the soil, often due to the inadequate disposal of human and animal waste, and the use of sewage sludge or contaminated water for agricultural irrigation [3]. Intermediate hosts such as cattle and pigs can ingest *Taenia* eggs, leading to cysticercosis, a tissue infection that affects the muscles, skin, eyes, and the nervous system [4].

Humans typically become infected with *Taenia* by eating undercooked or raw beef and pork meat from infected animals. In the human intestine, the larvae develop into adult tapeworms, causing taeniasis, a disease with significant human and veterinary health implications [5,6]. The disease also imposes an economic burden, including treatment costs, lost productivity, and the condemnation of infected meat [1]. Taeniasis is particularly prevalent in low- and middle-income countries in Latin America, Asia, and Africa, where inadequate sanitation, poor meat inspection practices, and limited access to proper cooking facilities are common [6,7]. An estimated 40–60 million people worldwide are affected by the disease [8].

Taeniasis treatment includes antiparasitic drugs such as niclosamide for *Taenia solium* [9] and combined with praziquantel for *Taenia saginata* [10]. The latter would act by opening calcium channels in the tapeworm’s membrane, causing an influx of calcium ions, leading to uncontrolled muscle contraction and paralysis [11]. Moreover, praziquantel in combination with corticosteroids is also effective against neurocysticercosis caused by *T. solium* [12]. In addition, fenbendazole and mebendazole have been successfully used for the treatment of cysticercosis in pigs in combination with laxatives such as magnesium sulphate for the expulsion of dead worms [1]. Although the drugs currently used for the treatment of taeniasis and neurocysticercosis are effective, their judicious administration in terms of the dose and duration of treatment is important. Frequent use of the same molecules has been reported as one of the factors contributing to the emergence of helminth resistance to different groups of available anthelmintics [13]. This resistance highlights the urgent need to develop new alternative agents [14].

Traditional medicine, especially herbal remedies, is the first line of treatment [15] for several types of infectious diseases [16] in African communities, especially in Togo [17]. Some herbal medicines have been reported to have hypoglycemic [18], antihypertensive [19], antimalarial [20], and antimicrobial properties [21], as well as others such as antidiabetic, anticancer, anti-inflammatory, and antioxidant activity and hepatoprotective and neuroprotective effects [22].

Numerous studies have demonstrated the anthelmintic potential of many medicinal plants against different worms such as *Haemonchus contortus* [23] and *Heligmosomoides bakeri* [24]. In recent years, the efficacy of plants as anthelmintics has been evaluated in several readily available experimental models such as *Caenorhabditis elegans* [25,26]; *Haemonchus contortus* [27]; *Eisenia fetida* [28]; *Trypanosoma brucei brucei*; and *Leishmania tarentolae* [29]. One study has shown that the essential oil of *Thymus bovei* had a stronger anthelmintic effect on *Pheretima posthuma* than piperazine citrate, the reference drug that was used [30]. In addition, the effectiveness of plants as anthelmintics against *Taenia* species commonly found in domestic animals such as cattle, pigs, goats, and sheep has been investigated in some studies. This is the case of a study which evaluated the anthelmintic properties of *Gongronema latifolium*, *Piper guineense*, and *Ocimum gratissimum* on bovine feces and demonstrated significant efficacy against *Taenia saginata* eggs [31].

In Togo, our previous research has identified *Khaya senegalensis*, *Aframomum melegueta*, and *Xylopia aethiopica* as plants traditionally used to treat helminth infections [32]. In addition, these plants have demonstrated anti-inflammatory activity; they are anti-Th17-Th2 in subjects with hyperreactive onchocerciasis [33]. Our tests also revealed that extracts from *A. melegueta* and *X. aethiopica* exhibit no toxic effects at therapeutic doses, supporting their traditional use and underscoring the need for further scientific validation [34].

This study aimed to investigate the anthelmintic activity of fractions derived from hydroethanolic extracts of *A. melegueta* and *X. aethiopica* against adult *Taenia* worms.

## 2. Results

### 2.1. Bio-Guided Extraction and Partitioning

The bio-guided extraction and fractionation of hydroethanolic extracts from *A. melegueta* (AM) and *X. aethiopica* (XA) yielded three fractions for each plant. Both plants produced an aqueous fraction (AF) and an ether fraction (EF). In addition, *A. melegueta* produced a dichloromethane fraction (DCMF), while *X. aethiopica* produced a chloroform fraction (CF).

### 2.2. Anthelmintic Activity of AM and XA Fractions on Taenia spp. Worms

**Figure 1:**
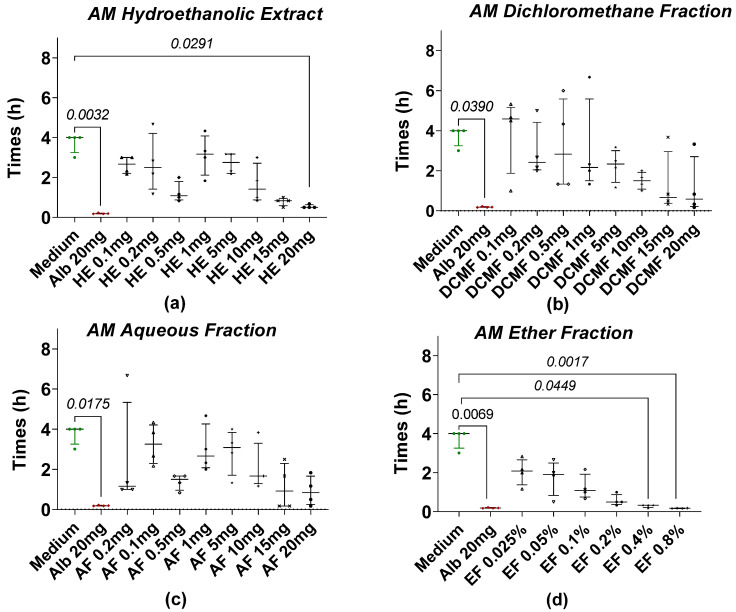
Loss of motility of the tapeworm in the presence of AM extract and fractions. (**a**) The hydroethanolic extract (HE), (**b**) the dichloromethane fraction (DCMF), (**c**) the aqueous fraction (AF), and (**d**) the ether fraction (EF). Alb refers to Albendazole. The green dots represent the negative control (medium), the red dots represent the reference drug (albendazole), and the black dots represent the different concentrations of plant extracts. Data are expressed as the median, and each point represents the mean time of loss of motility from 4 independent experiments with 5 worms per experiment. Statistical analysis was performed using the Kruskal–Wallis and Dunn’s post hoc tests.

The anthelmintic activity of hydroethanolic extracts (HEs) and their fractions (DCMF, CF, AF, EF) from AM and XA was evaluated using *Taenia* spp. worms. All of the tested fractions induced a loss of motility in the worms. The time required for worm paralysis after treatment with AM fractions at different concentrations is shown in Figure 1. Among the treatments, 20 mg/mL of AM hydroethanolic extract (Figure 1a) showed the most potent anthelmintic effects. Statistical analysis revealed a significant difference between the hydroethanolic extract of AM (Figure 1a) at a concentration of 20 mg/mL and the negative control, also called medium (RPMI 1640 + 10% FBS). In contrast, no significant differences were observed for the dichloromethane fraction (Figure 1b) and the medium at any concentration. Similarly, a comparison of the medium with different concentrations of the aqueous fraction (Figure 1c) showed no significant differences. However, the ether fraction (Figure 1d) showed significant differences compared to the medium at concentrations of 0.4% and 0.8%. Notably, after a median incubation time of 4 h, all worms in the negative control group (RPMI 1640 + 10% FBS) lost their motility. All fractions were tested in parallel with the same negative control. This experiment was repeated four times.

**Figure 2:**
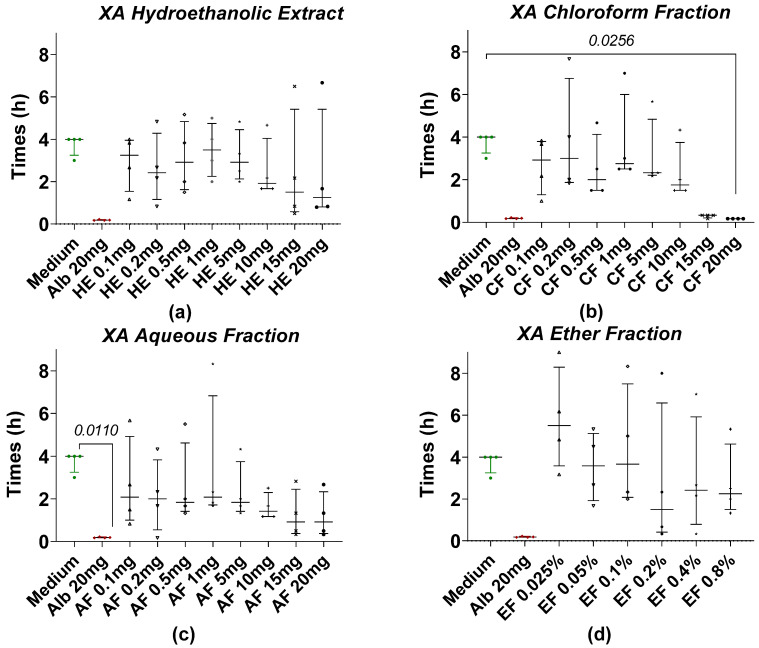
Loss of motility of the tapeworm in the presence of XA extract and fractions. (**a**) The hydroethanolic extract (HE), (**b**) the chloroform fraction (CF), (**c**) the aqueous fraction (AF), and (**d**) the ether fraction (EF). Alb refers to Albendazole. The green dots represent the negative control (medium), the red dots represent the reference drug (albendazole), and the black dots represent the different concentrations of plant extracts. Data are expressed as the median, and each point represents the mean time of loss of motility from 4 independent experiments with 5 worms per experiment. Statistical analysis was performed using the Kruskal–Wallis and Dunn’s post hoc tests.

Figure 2 shows the time taken for *Taenia* worms to lose motility when treated with hydroethanolic extract (HE) and fractions of *X. aethiopica* (XA) at different concentrations. After 4 h of incubation, most of the worms in the treatment groups were paralyzed at all concentrations of HE and fractions, while the worms in the negative control or medium (RPMI 1640 + 10% FBS) group remained motile. The most effective anthelmintic activity was observed at a concentration of 20 mg/mL of the XA chloroform fraction (Figure 2b) with a motility loss time comparable to the reference drug albendazole. Statistical analysis revealed a significant difference when we compared the negative control with the chloroform fraction at concentrations of 20 mg/mL. However, no significant differences were observed between the HE (Figure 2a), AF (Figure 2c), and EF (Figure 2d) of XA.

### 2.3. Anthelmintic Activity of HE and Fractions of AM and XA on Taenia Cells

To discover if our plant extracts act in the same way on the cells as on the whole worms, we treated the worms with the plant extract before and after isolating their cells with collagenase to analyze the percentage of dead cells by PI staining.

#### 2.3.1. *Taenia* Worm Cells’ Extraction Before Treatment with Extracts and PI Staining

**Figure 3:**
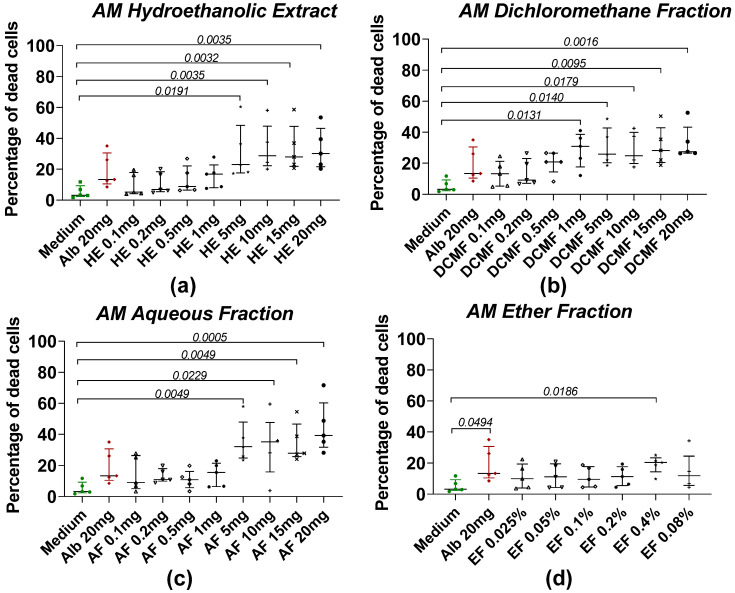
Induction of worm cell death by different fractions of AM. (**a**) The hydroethanolic extract (HE), (**b**) the dichloromethane fraction (DCMF), (**c**) the aqueous fraction (AF), and (**d**) the ether fraction (EF). Alb refers to Albendazole. The green dots represent the negative control (medium), the red dots represent the reference drug (albendazole), and the black dots represent the different concentrations of plant extracts. Data were expressed as the median, and each point represents the percentage of cell death of one worm. The experiment was performed on 5 worms. Statistical analysis was performed using the Kruskal–Wallis and Dunn’s multiple comparison post hoc test.

**Figure 4:**
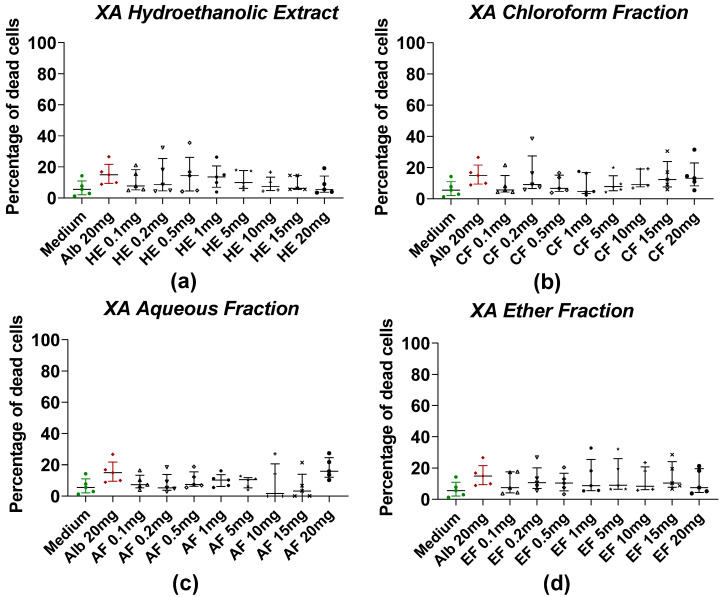
Induction of worm cell death by different fractions of XA. (**a**) The hydroethanolic extract (HE), (**b**) the chloroform fraction (CF), (**c**) the aqueous fraction (AF), and (**d**) the ether fraction (EF). Alb refers to Albendazole. The green dots represent the negative control (medium), the red dots represent the reference drug (albendazole), and the black dots represent the different concentrations of plant extracts. Data were expressed as the median, and each point represents the percentage of cell death of one worm. The experiment was performed on 5 worms. Statistical analysis was performed using the Kruskal–Wallis and Dunn’s multiple comparison post hoc test.

The effects of HE and fractions of AM and XA on *Taenia* cells obtained by enzymatic digestion with collagenase after treatment with plant extract are shown in Figure 3 and Figure 4, respectively. For AM, the mortality of *Taenia* cells under the effect of hydroethanolic extract (Figure 3a), dichloromethane (Figure 3b), and aqueous fractions (Figure 3c) was dose-dependent. Significant differences were observed between the medium and the HE, DCMF, and AF at concentrations of 5, 10, 15, and 20 mg/mL, whereas the EF (Figure 3d) showed significance only at 0.4%. For *XA*, the hydroethanolic extract (Figure 4a), the chloroform fraction (Figure 4b), the aqueous fraction (Figure 4c), and the ether fraction (Figure 4d) showed no significant differences compared to the negative control (medium).

#### 2.3.2. Treatment of *Taenia* Worms with Extracts Before Cell Extraction and PI Staining

**Figure 5:**
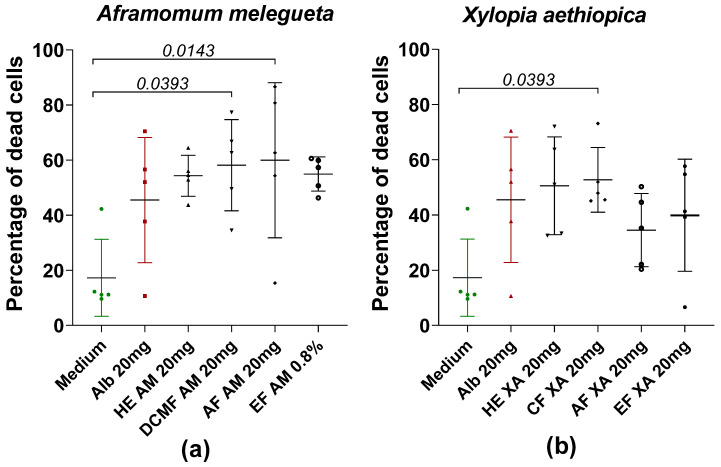
Percentage of dead cells after loss of motility of worms in the presence or absence of hydroethanolic extract (HE) and fractions of AM (**a**) and XA (**b**) at a concentration of 20 mg/mL. Alb refers to Albendazole. The green dots represent the negative control (medium), the red dots represent the reference drug (albendazole), and the black dots represent the different concentrations of plant extracts. Data are expressed as the median, and each point represents the percentage of cell death of one worm. The experiment involved 5 worms. Statistical analysis was conducted using the Kruskal–Wallis and Dunn’s multiple comparison.

Figure 5 shows the percentage of dead cells after the loss of motility of worms treated with HE and fractions of AM and XA at a concentration of 20 mg/mL. In general, all of the fractions induce cell death, highlighting that the fractions actually kill the worms and do not just cause starvation, emphasizing that the herbal remedies work and are an option for novel drugs. Furthermore, for AM, the dichloromethane and aqueous fractions showed significant differences compared to the medium (Figure 5a). For XA, only CF showed significant differences when it was compared to the medium (Figure 5b).

### 2.4. Irreversibility of the Effect of AM and XA Fractions on Taenia spp. Worms

To demonstrate the irreversibility of the anthelmintic activity of the AM and XA extracts and fractions, additional tests were performed using the most effective concentrations: 10, 15, and 20 mg/mL for HE, DCMF, CF, and AF, and 0.2%, 0.4%, and 0.8% for EF. Table 1 summarizes the times at which *Taenia* worms remained paralyzed after being exposed to the hydroethanolic extract (HE) and fractions (DCMF, CF, AF, and EF) of AM and XA and then placed in medium. The main goal of this test was to highlight that most of the worms were paralyzed (did not wake up) by our plant extracts and fractions.

In AM, when worms that had lost motility in the HE (all concentrations tested) were transferred to the medium (RPMI + 10% FBS), they showed no revival of motility. Similarly, no revival was observed in worms treated with the dichloromethane fraction (15 and 20 mg/mL) and the ether fraction (0.2, 0.4, and 0.8%), and they were transferred to the medium after paralysis. Regarding XA, worms treated at a concentration of 10 and 15 mg/mL of the HE did not recover their motility in the medium. Also, no revival was observed for worms treated with the chloroform fraction (15 and 20 mg/mL). As for the ether fraction, no revival was observed in worms treated with different concentrations and then placed in the medium after paralysis.

## 3. Discussion

Resistance to anthelmintics is a major public health problem. Several cases of resistance have been reported for helminthiasis caused by hookworm, ascariasis, and schistosomiasis [35,36]. Although the resistance reported for taeniasis is very rare, resistance remains a threat and thus requires the development of alternative treatments [37].

Plants have long been recognized as valuable sources of bioactive molecules to treat various diseases. Since then, numerous studies have investigated their biological activities against parasites, especially helminths [38,39]. Various worm species were used to evaluate the anthelmintic potential of plant extracts, as in a study by Buza et al. that demonstrated the anthelmintic activity of six aqueous medicinal plant extracts against donkey strongyles [40]. In another study, *Lumbricus terretris* worms were used to test the anthelmintic activity of aqueous and ethanolic extracts of *Vernonia amygdalina* and *Alstonia boonei* [41].

The present study is the first to evaluate the anthelmintic activity of two hydroethanolic extracts and six fractions derived from *A. melegueta* (AM) and *X. aethiopica* (XA) against whole worms and cells of *Taenia* spp in vitro. Our results show that all of the extracts and fractions of AM and XA have anthelmintic activity against *Taenia* spp. at both the tissue and cellular levels, with AM having a more pronounced effect on cells than XA.

These results highlight the potential of plant-based remedies as novel anthelmintics [42].

The effectiveness of an anthelmintic drug often varies depending on the developmental stage of the target parasite. Many drugs are effective only against larval stages [43], and those that target larvae may fail to act on adult worms.

For AM, the hydroethanolic extract (HE) and ether fraction (EF) showed significant anthelmintic effects. All worms were immobilized or killed within 9 h, contrasting with the 120 h timeframe reported by Mengome et al. on *Loa loa* microfilariae [22]. This difference likely stems from variations in parasite species, the concentrations used, and experimental conditions. While studies like those of Lakshmi et al. [23] emphasize the influence of methodology on the outcomes, our findings suggest that HE and EF are promising candidates for further studies. In contrast, only the chloroform fraction of XA demonstrated significant activity against *Taenia* spp. This aligns with previous reports of XA’s efficacy against parasitic infections [44]. The observed activity of XA may be attributed to its rich phytochemical composition, particularly alkaloids and terpenoids, which have documented anthelmintic effects [45]. These compounds likely disrupt cellular metabolism, leading to parasite death [46].

The better activity of AM compared to XA could be explained by the difference in phytochemical composition and therefore their different mode of action. Our previous study on these plants had shown that the hydroethanolic extracts of AM were more concentrated in polyphenols and flavonoids than XA [34].

Flavonoids, a diverse group of plant-derived polyphenolic compounds, have demonstrated significant anthelmintic activity against several parasitic worms. Their mechanisms of action are diverse, targeting different stages of the parasite life cycle and influencing different physiological processes. Some flavonoids have been shown to affect the neuromuscular system of worms, leading to reduced motility and subsequent paralysis. For example, quercetin and naringenin have been shown to significantly inhibit the motility of liver flukes such as *Opisthorchis felineus*, suggesting interference with neuromuscular function [47]. Flavonoids are thought to act synergistically with other phytochemicals to enhance anthelmintic efficacy. In *Pithecellobium dulce*, the combination of flavonoids such as kaempferol and quercetin with phenolic acids resulted in a potent ovicidal effect against *Haemonchus contortus*, indicating that flavonoids contribute significantly to the overall anthelmintic activity of plant extracts.

Irreversibility tests showed that some extracts were parasiticidal, irreversibly damaging worms, while others showed parasitostatic, temporarily paralyzing them. This dual activity underscores the importance of selecting appropriate fractions for targeted applications. For example, parasiticidal effects that inhibit essential worm metabolism and cause irreversible damage are desirable for achieving complete eradication in therapeutic applications [48,49].

Our cell viability assays using propidium iodide (PI) staining confirmed the dose-dependent anthelmintic activity of AM, with HE inducing higher cell mortality than albendazole. Notably, the hydroethanolic extract showed a synergistic action of its bioactive components, which may enhance its overall efficacy. However, further studies are required to isolate and characterize these components to identify the most active biomolecules.

A key observation was the higher mortality rate of extracted cells after the exposure of worms to the extracts compared to direct application on cells. For example, at 20 mg/mL, HE and AM fractions achieved mortality rates above 50% when acting extracellularly, compared to rates below 45% in direct cellular assays. This suggests that the extracts are more effective on extracellular targets, possibly by disrupting worm membranes and cellular components. These results are consistent with previous studies emphasizing the extracellular activity of flavonoids, a major component of AM. Considering the observed toxicity of the ether fraction (EF) in previous experiments, the evaluation of less toxic solvents should be prioritized in future research.

Hydroethanolic extracts, which have shown significant efficacy with potentially safer profiles, may be more suitable for subsequent animal studies. This approach is consistent with the need to balance efficacy and safety in the development of plant-based anthelmintics. In addition, according to the results obtained for the irreversibility test of the anthelmintic activity of our extracts, the hydroethanolic extracts and the ethereal fractions of AM and XA would contain biomolecules that would irreversibly paralyze *Taenia* worms. Since the main objective of our study was to find bioactive compounds, it would be more appropriate to isolate bioactive molecules directly from hydroethanolic extracts to implement new anthelmintic drugs.

## 4. Materials and Methods

### 4.1. Plant Material

**Figure 6:**
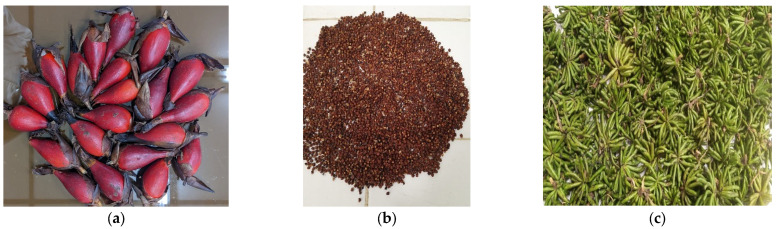
Plant material used in this study: (**a**) fruits of *Aframomum melegueta* K. Schum.; (**b**) seeds of *Aframomum melegueta* K. Schum.; (**c**) fruits of *Xylopia aethiopica* (Dunal) A. Rich.

The plant material used in this study consisted of the seeds of *A. melegueta* K. Schum. and the fruits of *X. aethiopica* (Dunal) A. Rich. (Figure 6). Both are spices with a rich history of culinary and medicinal use in West Africa and are recognized for their therapeutic properties [50,51].

The fruits of *A. melegueta* and *X. aethiopica* were collected, and botanical verification was carried out at the Botanical Laboratory of the Faculty of Science at the University of Lomé. The identification process was carried out using the International Plant Names Index (IPNI) to confirm the taxonomy of the plants. The voucher specimen was deposited at the Herbarium of the University of Lomé. The chemical characterization of the plants was previously described [34].

### 4.2. Plant Extraction

Fresh *A. melegueta* seeds and *X. aethiopica* fruits were washed and air-dried for two months at room temperature (18–25 °C) under controlled laboratory conditions and then crushed. The extraction of plant materials was performed by percolation. A total of 500 g of dried powdered seeds of *A. melegueta* and fruits of *X. aethiopica* was processed with 2 L of a 70% ethanol–water solution (70:30) for 72 h, with stirring every 24 h. After the first extraction, the filtrate was collected, and another 2 L of the ethanol–water solution was added to the remaining residue to repeat the process. The resulting extract was subsequently filtered through cotton and then through 11 µm Whatman No. 1 paper (Sigma-Aldrich, St. Louis, MO, USA) to ensure purity.

To obtain the hydroethanolic extract, a portion of the filtrate was evaporated to dryness at 50 °C under reduced pressure using a rotary evaporator (Buchi, Flawil, Switzerland). The remaining filtrate was partially evaporated to remove ethanol, leaving an aqueous extract.

### 4.3. Bio-Guided Partition

Bio-guided partition was carried out on the aqueous extract according to the modified method of Dosso et al. (2017) [52]. In our case, the solvents with increasing polarity used are petroleum ether, dichloromethane (for AM), and chloroform (for XA).

Equal volumes of the aqueous fraction and petroleum ether (Park Scientific Limited, Northampton, UK) (1:1) were mixed, vigorously homogenized, and allowed to decant, resulting in two distinct layers: an organic phase (ether fraction) and an aqueous phase. The aqueous phase was further partitioned with dichloromethane or chloroform (Chemicals & Metals Co, Gyeonggi, Republic of Korea). After mixing and decanting, two layers were obtained: an organic phase (dichloromethane or chloroform fraction) and a final aqueous phase. The aqueous phase was freeze-dried at 50 °C to obtain the aqueous fraction.

### 4.4. Collection of Worms

*Taenia* spp. worms, approximately 0.5–2 m long and 0.5–1 cm wide, were collected from the gut of naturally infected cattle and sheep slaughtered at the “Office National des Abattoirs Frigorifiques” (ONAF) in Lomé, Togo.

**Figure 7:**
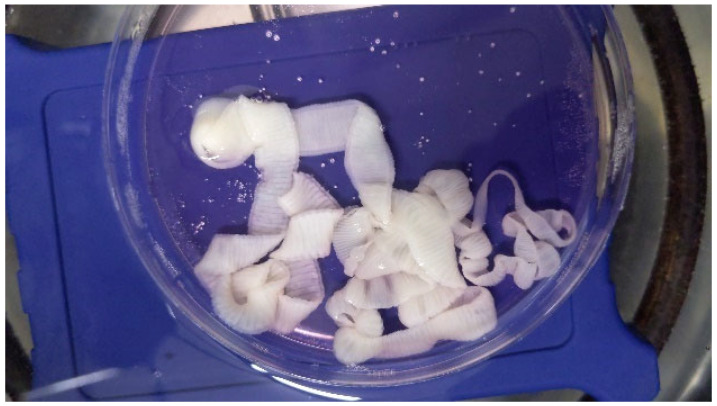
Tapeworm motility test in RPMI 1640 at 45 °C in a water bath.

Isotonic saline water (B. BRAUN, Melsungen, Germany) was used to wash all the worms and to remove all fecal material. The worms were immediately placed in RPMI 1640 medium (Gibco, Paisley, Scotland, UK) to maintain viability during transport to the laboratory. Slaughter of the animals began at 4 a.m. The time taken to wash all the intestines and collect the worms, if present, and then transport them to the laboratory varied between 4 and 6 h. However, once in the laboratory, the viability of worms was assessed by heating them in RPMI 1640 medium (Thermo Fisher Scientific, Waltham, MA, USA) at 45 °C in a water bath (Figure 7). A four-point scoring system ranging from 0 to 3 was used to describe the motility of worms in the different Petri dishes [53]. The vigorous movement of worms was scored at 3, the sluggish shaking of worms was scored at 2, the intermittent shaking of worms was scored at 1, and no movement (immotile) was scored at 0.

For the tests, worms that scored 3 were randomly divided into the different treatment groups.

### 4.5. Anthelmintic Activity on Taenia Worms

The in vitro anthelmintic tests of *A. melegueta* and *X. aethiopica* hydroethanolic-derived fractions on tapeworms were performed by a combination of the methods described by Akter et al. (2014) using five worms [54] and Lalthanpuii et al. (2020) for the motility test at 45 °C [55] with minor modifications. In our case, we modified by using *Taenia* spp. worms isolated on cattle and sheep and the reference drug albendazole at a concentration of 20 mg/mL.

Before treatment, stock solutions of the ether fractions (AM-EF and XA-FE) were obtained by dissolution in DMSO. Six dilutions of 0.025%, 0.05%, 0.1%, 0.2%, 0.4%, and 0.8% of each fraction were then prepared in 15 mL of RPMI medium supplemented with 10% heat-inactivated fetal bovine serum (FBS, PAN Biotech, Aidenbach, Germany). However, stock solutions of hydroethanolic extracts, dichloromethane, chloroform, and aqueous fractions (AM-HE, AM-DCMF, AM-AF, XA-HE, XA-CF, and XA-AF) were prepared in water. Eight dilutions (0.1, 0.2, 0.5, 1, 5, 10, 15 and 20 mg/mL) were made in 15 mL of RPMI medium supplemented with 10% heat-inactivated fetal bovine serum.

An anthelmintic assay was carried out by distributing 5 worms into 60 groups, corresponding to each extract concentration. RPMI medium supplemented with 10% heat-inactivated fetal bovine serum was used as the negative control. The reference drug albendazole (CPA GERERICS, Lomé, Togo) was also prepared in RPMI medium supplemented with 10% heat-inactivated fetal bovine serum at 20 mg/mL and served as the positive control. Petri dishes containing worms and plant extracts or controls were incubated at 37 °C, and motility was checked every 10 min after a brief exposure to 45 °C. The time of worm paralysis was recorded after confirming that the worms did not move when heated or when shaken vigorously. Each experiment was replicated four times.

### 4.6. Anthelmintic Activity on Taenia Cells

**Figure 8:**
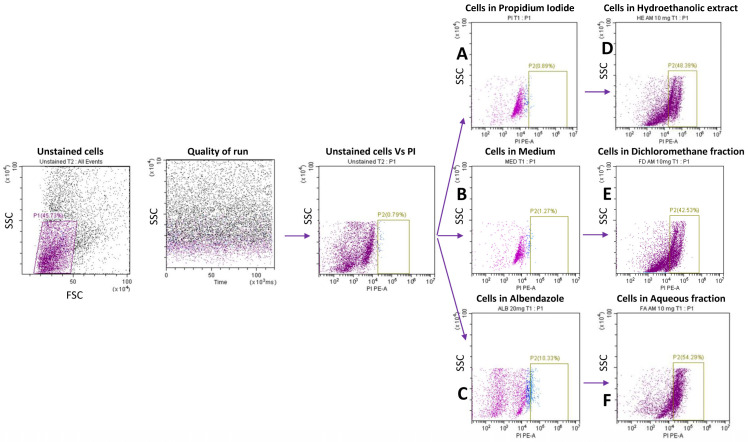
The anthelmintic effect of AM extract and fractions on Taenia cells (1 × 10^4^ cells/well) was left alone (Med) or stimulated with 20 mg/mL of ALB and 10 mg/mL of HE, FD, and FA for 12 h. Cells were stained with propidium iodide dye (PI) and acquired by flow cytometry. A: PI cells gate, B, C, D, E, and F are PI+ cells alone, in the presence of medium, albendazole, and 10 mg/mL of HE, FD, and FA, respectively.

To assess the effect of the extract on *Taenia* cells, we performed a propidium iodide (PI) staining on *Taenia* cells derived from dead worms (loss of motility) and on *Taenia* live cells after exposure to the plant compound. Briefly, adult worms were exposed to the extract, as described, and after loss of motility (12–18 h), the five worms were digested with 100 µL of collagenase (Roche Diagnostics, Mannheim, Germany) at a concentration of 2 mg/mL and incubated at 37 °C with 5% CO_2_ for 3–4 h. In addition, five live worms were digested with collagenase, as described, and the obtained cells were co-cultured with the extracts for 12–18 h. Cells from dead worms and the exposed cells were then stained with PI. The rate of cell cytotoxicity in both cases was compared. The gating strategy used is presented in Figure 8.

### 4.7. Propidium Iodide Staining of Taenia Cells

After the incubation period, the supernatant was carefully removed from each well, and the remaining cells were stained with propidium iodide (PI) (Avantor, Radnor, PA, USA) for flow cytometry analysis. After removing the supernatant from the wells, 200 µL of FACS buffer (PBS + 2% FBS) was added to each well. Cells were collected in hemolysis tubes and centrifuged at 1500 rpm for 5 min. The supernatant was discarded, and the cells were incubated with 100 µL of PI in the dark. After incubation for 15 min at 4 °C, the cells were washed again with 200 µL of FACS buffer, resuspended in another 100 µL of FACS buffer, and analyzed using a Cytoflex flow cytometer (Beckman Coulter, Brea, CA, USA). Data were processed using CytExpert 2.1 software (Beckman Coulter, Brea, CA, USA). The percentage of cells expressing propidium iodide (PI) was assessed. The gating strategy used is described in Figure 8.

### 4.8. Assessment of Motility Recovery of Taenia spp. Worms

**Figure 9:**
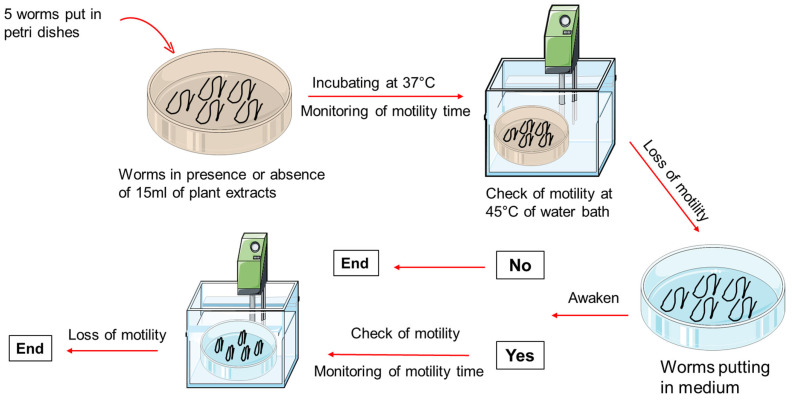
Schematic overview of the experimental design.

To determine whether the anthelmintic activity of the AM and XA fractions was irreversible, a separate set of experiments was performed using the most effective concentrations: 10, 15, and 20 mg/mL for HE, DCMF, CF, and AF, and 0.2%, 0.4%, and 0.8% for EF. After complete loss of motility (score 0) upon exposure to these concentrations, they were transferred back to the culture medium (RPMI 1640 + 10% FBS) alone and observed for any recovery of motility (Figure 9). This irreversibility test was performed to distinguish between the reversible and irreversible anthelmintic effects of the plant fractions tested.

### 4.9. Statistical Analysis

Statistical analysis of the data was performed using the GraphPad PRISM 8.0.2 software (GraphPad Software, Inc., La Jolla, CA, USA). A D’Agostino–Pearson normality test was used to test for the distribution of the values before testing for statistical significance between the groups. Since the variables were nonparametrically distributed, Kruskal–Wallis tests were performed to compare more than two treated groups with the control group. If the Kruskal–Wallis test was significant, Dunn’s multiple comparison post-test was performed to further compare the groups. Data were considered significant for *p*-values of 0.05 or less (* *p* < 0.05, ** *p* < 0.01, *** *p* < 0.001).

## 5. Conclusions

This study revealed that the HEs and fractions (EF and DCMF/CF) of each plant exhibited significant, dose-dependent anthelmintic activity against *Taenia* spp. In particular, AM-HE and XA-CF showed a high effectiveness at 20 mg/mL and AM-EF at 0.4% and 0.8%. These results underscore the potential of AM and XA extracts and fractions as alternative treatments for helminth infections and provide scientific evidence of the efficacy of these plant extracts. While their efficacy is promising, further research is needed to isolate active compounds, refine solvent selection, and evaluate safety profiles in animal models. By focusing on these aspects, future studies may pave the way for the development of effective herbal anthelmintics.

## Figures and Tables

**Table 1:** Time to recovery motility or not of *Taenia* worms in the medium after loss of motility in the HE and fractions (DCMF, CF, AF, and EF) of AM and XA.

Extracts	Concentrations (mg/mL)	Initial Time of Loss of Motility (h)	Transfer in Medium	
			Wake Up Time (h)	Paralysis Time (h)
Medium		4.82	-	-
*A. melegueta*				
HE	10	1.16	-	-
	15	1.06	-	-
	20	1	-	-
DCMF	10	1.28	2.11	6
	15	1.44	-	-
	20	0.94	-	-
AF	10	1.78	0.17	0.33
	15	1.72	0.17	0.50
	20	1.67	0.61	1.50
EF	0.2%	0.61	-	-
	0.4%	0.44	-	-
	0.8%	0.28	-	-
*X. aethiopica*				
HE	10	2.33	-	-
	15	2.17	-	-
	20	2	0.17	0.21
CF	10	3.22	-	-
	15	2.94	-	-
	20	2.89	0.56	2
AF	10	3.50	0.44	2
	15	3.22	0.5	2
	20	2.94	0.56	2
EF	0.2%	2.17	-	-
	0.4%	3	-	-
	0.8%	1.94	-	-

Abbreviations used: HE—hydroethanolic extract; DCMF—dichloromethane fraction; AF—aqueous fraction; EF—ether fraction; CF—chloroform fraction. Post hoc test.

## Data Availability

The original contributions presented in this study are included in the article. Further inquiries can be directed to the corresponding authors.

## Abbreviations

AF	Aqueous fraction
AM	*Aframomum melegueta*
CF	Chloroform fraction
DCMF	Dichloromethane fraction
EF	Ether fraction
FBS	Fetal bovine serum
HE	Hydroethanolic extract
PBS	Phosphate-buffered saline
RPMI	Roswell Park Memorial Institute medium
XA	*Xylopia aethiopica*
